# In Situ Evaluation of the Self-Heating Effect in Resistance Temperature Sensors

**DOI:** 10.3390/s25113374

**Published:** 2025-05-27

**Authors:** Przemysław Otomański, Eligiusz Pawłowski, Anna Szlachta

**Affiliations:** 1 Institute of Electrical Engineering and Electronics, Faculty of Control, Robotics and Electrical Engineering, Poznan University of Technology, Piotrowo Street 3A, 60-965 Poznan, Poland; 2 Department of Automation and Metrology, Faculty of Electrical Engineering and Computer Science, Lublin University of Technology, Nadbystrzycka Street 38A, 20-618 Lublin, Poland; e.pawlowski@pollub.pl; 3 Department of Metrology and Measurement Systems, Faculty of Electrical and Computer Engineering, Rzeszow University of Technology, W. Pola Street 2, 35-959 Rzeszow, Poland; annasz@prz.edu.pl

**Keywords:** current–voltage characteristics, industrial platinum resistance thermometers, self-heating, sensor systems and applications, temperature sensors, temperature measurement, thermal management of electronics, thermal resistance, uncertainty

## Abstract

**Highlights:**

**What are the main findings?**

- The self-heating effect of a resistance sensor depends on its thermal resistance, which should be determined in situ.
- The thermal resistance of a sensor can be determined as the slope of the temperature characteristic as a function of heating power.

**What is the implication of the main findings?**

- The internal thermal resistance of the sensor depends on its design, and the external thermal resistance of the sensor is dependent on the heat transfer conditions between the sensor’s sheath and its environment.
- In situ testing of sensors allows the separation of self-heating due to internal and external effects.

**Abstract:**

This paper discusses the issue of the self-heating effect of resistance sensors during temperature measurement. The self-heating effect causes temperature measurement errors. The aim of this work was to develop a method for in situ assessment of the thermal resistance between a self-heating thermometer and its surrounding environment, the temperature of which is measured. The proposed method is used to assess the uncertainty resulting from the heat transfer from the thermometer to the surrounding environment, which allows increased measurement accuracy. The proposed method consists of experimental determination of the sensor’s temperature characteristics in relation to the heating power for different values of the measuring current. Sample measurements were carried out on a representative group of resistance temperature sensors. The relationship of the internal thermal resistance to the type of sensor design and the relationship of the external resistance to the ambient conditions were demonstrated. The developed method allows the appropriate measuring current of the resistance temperature sensor to be selected according to its design, the mounting method, and the environmental conditions, which ensures that measurement errors are maintained at an appropriately low level.

## 1. Introduction

Temperature is one of the basic parameters that is often measured in practice. Various sensors, transducers, and devices are used to measure temperature. Some of the most commonly used sensors are resistance sensors including metal and semiconductor sensors (thermistors). Typical metals used in these types of sensors are platinum, copper, and nickel.

The issue of resistance temperature sensors RTD is covered in a number of normative documents [1,2,3,4,5,6,7]. In practice, there are two types of resistance sensors; these are standard platinum resistance thermometers with high stability (SPRTs) [2] and industrial platinum resistance thermometers (IPRTs) [3]. IPRT sensors are widely used in industrial electronics systems, but the self-heating effect of these sensors has been reported sporadically in the literature [8]. For this reason, the current study addresses this issue.

Guidelines [1] outline the issues related to the establishment of temperature scales today and in the future, as part of a document prepared on behalf of the Consultative Committee on Thermometry, discussing methods of implementing the International Temperature Scale. SPRTs calibrated at specific fixed points and using appropriate interpolation procedures [4] are used to reproduce the ITS-90 temperature scale in the temperature range between the triple equilibrium point of hydrogen (13.8033 K) and the freezing point of silver (1234.93 K) [1].

Platinum is one of the metals that is used in resistance thermometers. It is preferred because of its very wide temperature range, across which it shows good resistance to chemical and physical effects that affect the resistance-temperature characteristics of thermometers. The issues of self-heating and error correction for these platinum sensors used in relation to the ITS-90 scale have also been discussed. Typical SPRTs have a resistance of 25 Ω at the triple point of water (TPW) or in the range of 0.2 Ω to 2.5 Ω for sensors operating in the upper temperature range. For currently available SPRTs with a typical resistance of 25 Ω, with a recommended measurement current of 1 mA, the self-heating effect ranges from 0.2 mK to 4 mK [2].

IPRT measuring instruments use a single measuring current and therefore cannot measure the self-heating effect. During calibration, IPRT resistance measurements are normally taken at the same measuring current. Subsequent use of a calibrated thermometer requires the resistance to be measured with an instrument operating at the same measuring current as used during calibration. Manufacturers usually specify the self-heating coefficient of their IPRT sensors or probes, measured in a specific medium. The coefficient can be given in two forms: self-heating coefficient, in °C/mW, or dissipation constant, in mW/°C. For standard sensors, the self-heating coefficient takes values from 0.003 °C/mW to 0.25 °C/mW, depending on the sensor design, mounting method, and parameters of the medium in which the measurement is made [3]. It should be noted that the self-heating factor is equivalent to the concept of thermal resistance, which in the SI system has the unit K/W, but the more numerically practical unit is °C/mW.

A different approach is presented elsewhere [5], where the tolerance of the sensors is given according to the class. This standard distinguishes between wire and thin-film sensors. According to this standard, the tolerance for industrial sensors at 0 °C is from 0.1 °C to ±0.6 °C and at the end of the measurement range from ±0.36 °C to ±6.6 °C, depending on the tolerance class. The value of the measurement current should be selected as small enough so that the self-heating effect of the sensor does not exceed 25% of the tolerance value resulting from the declared tolerance class of the sensor. However, at the same time, a higher measuring current provides greater accuracy when measuring the sensor’s resistance. Therefore, selecting the optimal value of the measuring current requires knowledge of the thermal resistance of the sensor, which strongly depends on the way the sensor is mounted and the parameters of the surrounding medium [5].

The requirements for IPRTs are described in a number of standards, the most widely used of which are the European [5] and American [6,7] standards. In both standards [5,6], the same processing characteristics of IPRTs are defined, but the permissible sensor tolerances are defined differently and the method of testing the self-heating effect is described in different ways [5,7,9].

According to IEC 60751 [5], self-heating is the increase in temperature of a resistor in a thermometer due to the dissipated energy of the measurement current, while the self-heating coefficient is a coefficient with the dimension °C/mW specific to the resistor/thermometer and describing the increase in temperature of the resistor per unit of power dissipated. The self-heating coefficient should be assessed at a temperature of 0 °C to 30 °C in water flowing at a velocity of >0.2 m/s and/or in air flowing at a velocity of (3 ± 0.3) m/s. Self-heating under the above-mentioned conditions should not exceed 25% of the tolerance value of the declared tolerance class at the declared maximum test current. According to another standard [7], the self-heating coefficient should be evaluated at a temperature close to 25 °C in water flowing at a velocity of 1 m/s, or in air or other gas flowing at a velocity of 5 m/s ± 10%. The parameter that quantitatively describes the self-heating effect occurring with PRTs is thermal resistance. Its value depends on the design of the device in question (e.g., a resistance temperature sensor) and the environmental conditions (thermal conductivity, heat transfer coefficient, velocity of the medium, etc.).

Knowing the thermal resistance makes it possible to correct errors in the SPRT sensor resulting from its self-heating or to select the optimum conditions for its IPRT supply to minimize the values of these errors. Therefore, to correctly select the measurement current of the IPRT sensor, the in situ thermal resistance of the sensor must be determined. This coefficient is evaluated under the specific operating conditions of the resistor or thermometer to determine the medium, its flow conditions, and temperature.

## 2. State of the Art

A previous paper [10] investigated the phenomenon of self-heating of standard platinum resistance thermometers (SPRTs). Particular attention was paid to investigating the factors that contribute to the uncertainty assessment of the self-heating correction. A two-current self-heating correction method was used and additional correction methods were proposed, which consisted of optimal current selection and the use of more than two different currents. A reduction in the uncertainty of the self-heating correction from 0.04 to 0.01 mK was achieved. This decrease was not significant with regard to SPRT calibrations, but it may represent an improvement in the highest-accuracy measurements.

In the case of SPRT sensor measurements, the correction of the self-heating effect is carried out using two-current [11,12,13,14,15,16,17,18,19,20] or multi-current methods [11,12,13,17,20]. In another paper [11], the dependence of the random uncertainty of the corrected resistance on the ratio of measurement currents and the ratio of measurement times was investigated. It was assumed that the self-heating effect was proportional to the square of the current and that the uncertainty associated with the measured resistance varied inversely with the current and the square root of the measurement time.

In [12], it was shown that a 40% reduction in the uncertainty of the self-heating correction could be obtained by changing the conventional 1:$\sqrt{2}$ current ratio to 1:2. Further reductions were obtained by increasing the measurement time at the lower current and/or optimizing the allocation of time spent measuring at these two optimum currents, but with a diminishing return in either case. The two-current method uses two measurements of the sensor resistance *R*_1_ and *R*_2_ for two different current values *I*_1_ and *I*_2_ and then calculates the resistance *R*_0_ corresponding to the zero current value *I* = 0 mA at which the self-heating effect does not occur.

On the other hand, it was observed in other work [13] that the value of the self-heating correction increases as the thermal conductivity of the SPRT shell material and the gas filling it decreases. Self-heating correction was found to vary with temperature, so it should be estimated for all measured temperatures and corrected accordingly. Therefore, for accurate and precise temperature measurements, the effect of self-heating must always be corrected against the measured resistance value at all temperatures.

A further paper [14] investigated different methods to improve extrapolation by optimizing the choice of two currents and investigating whether measurement at more than two currents would reduce extrapolation uncertainty and, if so, how many currents and which extrapolation scheme to use. Extrapolation schemes using more than two currents were investigated using the Monte Carlo method. The most effective approach was found to be to take extra time to make additional measurements at the lower of the two currents, in a ratio of 0.5:1. The Monte Carlo method was used in another paper [15] to determine the temperature measurement uncertainty of an intelligent temperature transducer.

In high-accuracy measurements, the uncertainty of the self-heating correction obtained by the basic two-current method discussed previously may not be sufficient. A previous paper [16] presents more advanced methods for self-heating correction based on the use of more than two different currents. The use of more than three currents resulted in only a small additional improvement in uncertainty.

In an article [17], a model measurement for the thermal resistance of a sensor installation using the two-current method was presented. On the basis of the experimental results, the uncertainty of the thermal resistance measurement was analyzed and calculated. The increase in the sensor excitation current was found to lead to a reduction in the standard deviation of the temperature measurement.

In a different paper [18], the effect of self-heating was investigated for an SPRT measuring the triple point of water. Different SPRT resistance values were measured for carrier currents from 0.1 to 2 mA. However, the measurement results indicated that for currents below 0.5 mA, the SPRT resistance values were unstable.

It is worth mentioning that the approach to eliminating the self-heating effect for industrial IPRT sensors is somewhat different from that for standard SPRT sensors. For IPRT sensors, the operating conditions are important.

An article [19] presented an experimental method and algorithm for determining the self-heating of an industrial platinum resistance thermometer (IPRT) when the instability of the temperature of the medium of interest prevents the accurate determination of self-heating using standard methods. The method was used to determine the self-heating of a 100 Ω industrial IPRT designed to measure the air temperature inside the saturation chamber of a primary dew point/frost generator. Instead of a single measurement at each current, a sequence of temperature measurements was recorded, and the currents were switched after each measurement. In general, in environments with less stable temperatures, the number of repetitions should be higher than in environments with higher stability.

PRTs are common types of thermometers in AWS automatic weather stations, as presented in the work of [20,21]. The potential heating comes not only from the resistance temperature sensor itself but also from the accompanying electronics and sensors (humidity, etc.). In order to minimize the effect of self-heating, the lower limits of the electronic supply voltage declared by the manufacturer should be used in the AWS, or alternatively, the exposure time should be minimized [20].

Calibrations in air are associated with higher uncertainties than calibrations in liquid and for this reason, they are more often performed in a liquid bath, resulting in an additional source of uncertainty. In this situation, the self-heating error is included in the calibration corrections [21].

In previous works [10,11,12,13,14,16,17,18,19,20,21], the self-heating effect has been considered as an undesirable phenomenon, resulting in temperature measurement errors. On the other hand, however, since the self-heating effect depends not only on the sensor design but also on the flow velocity of the medium in which it is placed, it is possible to exploit this effect for the design of flow sensors. In this case, the self-heating phenomenon of PRT is therefore desirable and is used in the measurement of physical quantities other than temperature.

Several papers [1,2,3,4,10,11,12,13,14,16,17,18] present methods to correct the self-heating effect during the calibration of SPRT sensors. For IPRT sensors, researchers [5,6,7,9,19,20,21] have provided recommendations for the selected measurement current, ensuring an acceptable level of error resulting from the self-heating effect. However, no discussion of methods enabling the investigation of the self-heating effect under operating conditions is given, which is necessary for the proper selection of the measurement current. For this, it is necessary to know the thermal resistance of the sensor determined in situ.

This paper proposes a method for in situ testing of the self-heating effect of IPRT sensors and determination of their thermal resistance. The measurement system designed for this purpose is presented and the method of determining the thermal resistance implemented in the LabVIEW environment is discussed. The main objective of this work is to provide a method for assessing the thermal resistance between a self-heating thermometer and the surrounding environment, to enable an evaluation of the uncertainty caused by heat transfer from the thermometer to the environment. A linear least-squares approximation of the experimentally determined temperature dependence of the sensor on the heating power was used. The developed method enables static and dynamic testing, which makes it possible to determine the internal and external thermal resistance of sensors. Several examples of resistance temperature sensors, including IPRTs, were investigated. Thermal resistance values obtained from measurements of these sensors under different ambient conditions are given.

## 3. Research Method

### 3.1. Characteristics of Platinum Sensors

Among RTDs (resistance temperature detectors), the two basic types that can be distinguished are metallic and semiconductor (thermistors). Metal resistance temperature sensors exploit the phenomenon of how the resistance of metals changes as a function of temperature. The most commonly used sensors in practice are platinum sensors, whose characteristics and metrological properties are summarized in IEC 60751:2022-11 [5]. For this type of sensor, the dependence of the sensor resistance as a function of temperature, according to [5], can be described by the relation in the positive temperature range [4,22]:

(1) $$R_{T}=R_{0}\left(1+AT+BT^{2}\right),$$

where *R_T_* is the resistance of the sensor at the test temperature, *R*_0_ is the resistance of the sensor at 0 °C, *A* = 3.9083 × 10^−3^ °C^−1^, *B* = −5.775 × 10^−7^ °C^−2^ [5].

In this study, it was necessary to convert the resistance of the sensor to temperature to the following form, according to the transformed Formula (1) [6,21]:

(2) $$T=\frac{-A+\sqrt{A^{2}+4B\left(R_{T}/R_{0}-1\right)}}{2B}.$$

It should be noted that in older versions of the document [6], Equation (2) contained an error, which has been corrected in the current version of the standard.

### 3.2. Principle for Determining Thermal Resistance of the Sensor

Temperature practically affects the operation of all technical devices. It depends on the power provided in the device in question and the thermal resistance between the environment and that device. For temperature sensors, this effect results in temperature measurement errors, as described in Section 2. This effect is also considered for a number of semiconductor devices (transistors, diodes, integrated circuits, LEDs, etc.) where an increase in temperature adversely affects their performance; these factors include efficiency, gain, losses, durability, reliability, and others. It therefore seems that the method for determining thermal resistance proposed in this paper may have wider application than only for resistance temperature sensors.

The proposed method for determining thermal resistance involves experimentally determining the temperature characteristics of an object as a function of the power dissipated in it. Thermal resistance is the slope of a straight line that approximates the obtained characteristic.

Thermal resistance can be considered in the same way as electrical resistance. The basic formulas for thermal calculations can be treated in the same way as Ohm’s law.

**Figure 1:**
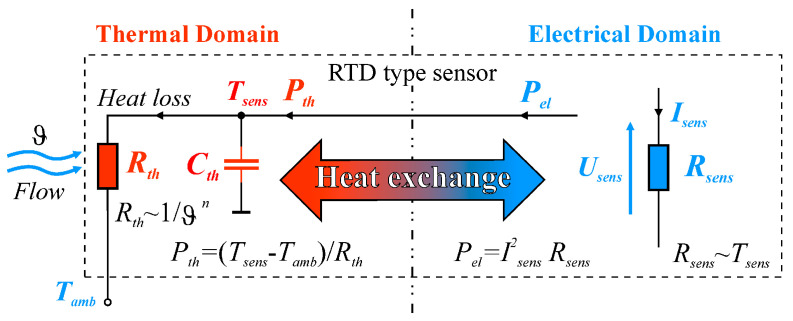
The principle of heat exchange between RTD and the environment.

The similarity between thermal and electrical parameters is particularly applicable to RTD-type sensors, which operate at the interface between two areas: electrical phenomena and thermal phenomena (Figure 1). Current *I*_sens_ flowing through the sensor’s resistance *R*_sens_ generates electric power *P_el_* = *I*^2^_sens_*R*_sens_. The power *P_el_* generated in the sensor increases the sensor’s temperature *T*_sens_ and is transferred by convection to its surrounding fluid flowing at rate ϑ, with ambient temperature *T*_amb_. The dynamic parameters of the sensor are determined by the heat capacity *C_th_*. They depend on the mass of the sensor and the specific heat of the material from which it is made. The product of the thermal resistance *R_th_* and the heat capacity *C_th_* of the sensor determines its thermal time constant.

The thermal power *P_th_* transferred from the sensor to its surroundings is as follows:

(3) $$P_{th}=h\cdot S_{\mathrm{sens}}\left(T_{\mathrm{sens}}-T_{\mathrm{amb}}\right),$$

where *h* is the heat transfer coefficient, *S*_sens_ is the sensor’s area.

The *h* coefficient describes the heat exchange between the sensor sheath and its surroundings. It depends on two components. The first component depends on the heat transfer between the surface of the sensor sheath and the layer of immediately adjacent fluid. The properties of the sensor surface and the surrounding fluid are important here. The second component depends on the thermal resistance of the surrounding fluid, which affects the rate of heat dissipation in the remaining volume of the fluid.

As originally proposed by King (1914), the heat transfer coefficient *h* varies with fluid velocity ϑ according to the following empirical relation:

(4) $$h=a+b\vartheta^{n}$$

where *a*, *b*, *n* are experimentally determined constants.

The equation is known informally as King’s empirical law. Constant *a* describes convection in motionless fluid (ϑ = 0), constant *b* specifies convection in flowing fluid. In a steady state, when the sensor’s temperature *T*_sens_ and fluid flow rate ϑ are constant over time, electric power *P_el_* supplied to the sensor is equal to thermal power *P_th_* dissipated to the surrounding area, stated as follows:

(5) $$P_{el}=P_{th}=S_{\mathrm{sens}}\left(a+b\vartheta^{n}\right)\left(T_{\mathrm{sens}}-T_{\mathrm{amb}}\right)$$

The ratio of the temperature difference *T*_sens_ − *T*_amb_ to power *P_th_* dissipated by the sensor to the surrounding area is defined as the sensor’s thermal resistance *R_th_*, as follows:

(6) $$R_{th}=\frac{T_{\mathrm{sens}}-T_{\mathrm{amb}}}{P_{th}}=\frac{1}{S_{\mathrm{sens}}\left(a+b\vartheta^{n}\right)}$$

Since *S*_sens_, *a*, *b*, *n* are constant, if we know the ambient temperature *T*_amb_ and electric power *P_el_* necessary to maintain the sensor’s temperature *T*_sens_, we can use Relation (5) to determine fluid flow rate ϑ. However, if the goal is to measure the temperature *T*_amb_ of the medium in which the sensor is placed, the result is a temperature measurement error Δ*T*_sens_, stated as follows:

(7) $$\Delta T_{\mathrm{sens}}=T_{\mathrm{sens}}-T_{\mathrm{amb}}=R_{th}P_{th}=R_{th}I_{\mathrm{sens}}^{2}R_{\mathrm{sens}}$$

By transforming Relation (3), we obtain a linear dependence of the sensor temperature *T_sens_* on the heating power *P_th_*, as follows:

(8) $$T_{\mathrm{sens}}=R_{th}P_{th}+T_{\mathrm{amb}}$$

By approximating the measurement results with a straight line in the form *y* = *a*_0_ *+ a*_1_*x*, we obtain the thermal resistance of the sensor *R_th_* = *a*_1_ and the ambient temperature *T*_amb_ = *a*_0_. Least-squares approximation was used.

**Figure 2:**
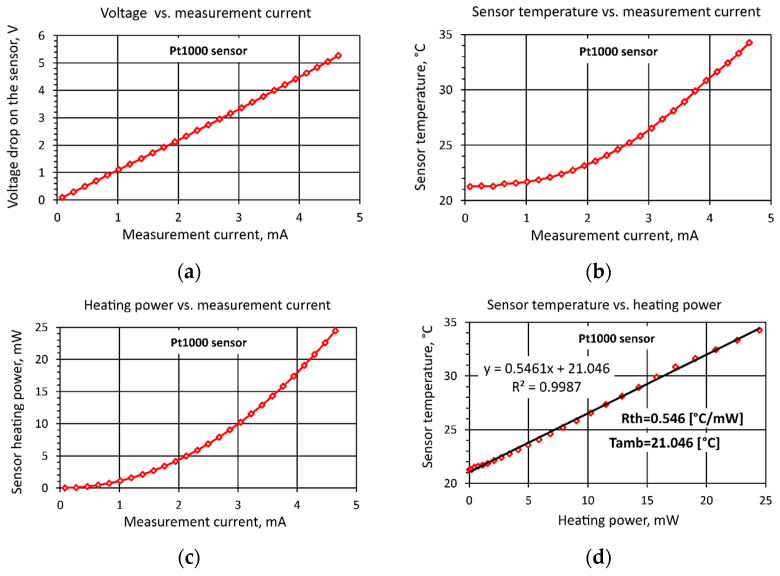
Diagrams for determining the thermal resistance of the sensor: (**a**) measured dependence of voltage drop on the sensor on measuring current (almost linear); (**b**) calculated dependence of sensor temperature on measuring current (almost quadratic); (**c**) calculated dependence of sensor heating power on measuring current (almost quadratic); (**d**) linear dependence of sensor temperature on heating power.

A practical implementation of the presented method is shown in Figure 2, using the example of a Pt1000 sensor, tested across a current range up to 5 mA. The first step is to measure the current-voltage characteristics [23] of the sensor under testing (Figure 2a). The next step is to calculate the resistance of the sensor from Ohm’s law and then use this to determine the temperature of the sensor from Equation (2). The determined characteristics of the sensor temperatures for different measurement current values are shown in Figure 2b.

The resistance of the sensor changes and therefore, the heating power is not exactly proportional to the square of the current, so this characteristic should not be approximated by a second-degree polynomial. The power emitted by the sensor is calculated as a function of current, taking into account the changing resistance of the sensor, as shown in Figure 2c. The last step of the proposed method is to represent the measurement results in terms of the dependence of the sensor temperature *T*_sens_ on the electrical power:

(9) $$P_{el}=I_{\mathrm{sens}}^{2}R$$

This dependence is then approximated by a straight line (8), as shown in Figure 2d. The directional coefficient of the straight line *y* = 0.5461*x* + 21.046 is the determined thermal resistance of the sensor *R_th_* = 0.546 °C/mW, and the free expression is equal to the ambient temperature *T*_amb_ = 21.046 °C.

### 3.3. Testing Other Sensors

In measurement practice, in addition to the SPRT [1,2,3,4,10,11,12,13,14,16,17,18] and IPRT [5,6,7,9,19,20,21] sensors described in the normative documents, metallic sensors made of copper, nickel, or Balco (nickel and iron alloys) are used. The processing equations for resistance metal sensors other than platinum are analogous to Equation (1). However, these equations differ in the degree of the polynomial and the values of the coefficients, depending on the metal used.

### 3.4. Method of Measuring Electrical Resistance of the Sensor

The method described in Section 3.2 for determining the thermal resistance of the sensor requires a circuit that allows the electrical resistance of the sensor to be measured for different values of current. In addition, the following values must be determined: the measurement current of the sensor *I*_sens_, the power dissipated in the sensor *P*_sens_*,* and the temperature of the sensor *T*_sens_. Such a system can be realized using two different measurement methods. The first method involves measuring the voltage drop across the resistance of the sensor under testing, using a suitable stable and regulated current source. The resistance of the sensor *R*_sens_ is calculated from Ohm’s law. The second method, which is ratiometric, involves measuring the voltage drops on the sensor resistance *R*_sens_ and on the reference resistor *R_N_* [24]. In the present work, the ratiometric method was used. The resistance value *R*_sens_ was calculated from the following formula:

(10) $$R_{\mathrm{sens}}=\frac{U_{\mathrm{sens}}}{U_{N}}R_{N}$$

where *U*_sens_ is the voltage measured at the sensor, *U_N_* is the voltage measured at the reference resistor, and *R_N_* is the nominal value of the reference resistor. The value of the sensor’s measurement current *I*_sens_ is calculated from the following formula:

(11) $$I_{\mathrm{sens}}=\frac{U_{N}}{R_{N}}.$$

The value of the power dissipated in the sensor *P*_sens_ under testing is calculated from the following formula:

(12) $$P_{\mathrm{sens}}=U_{\mathrm{sens}}I_{\mathrm{sens}}=\frac{U_{\mathrm{sens}}U_{N}}{R_{N}}.$$

The values determined from Equations (10)–(12) enable the characteristics shown in Figure 2 to be determined as described in Section 3.2 [23].

## 4. Results of Experimental Studies

### 4.1. Resistance Temperature Sensors Tested

The described method, LabVIEW (version 20.0f1) application, and measurement system were used to investigate the self-heating effect of selected IPRT sensors. The NI USB-6341 module from National Instruments (Austin, TX, USA) was used, containing 16-bit ADCs and DACs, providing an absolute voltage accuracy of 2.19 mV over a 10 V range (Table 1) [25].

The following typical examples of IPRT sensors were used for this study. Sensor number 1 was a thin film Pt1000 sensor on a ceramic substrate. Sensor number 2 was a Pt100 wire sensor in a cylindrical ceramic housing, while sensor number 3 was identical to 2, in an industrial steel housing. Sensor number 4 was a Pt500 wire sensor enclosed in a waterproof steel housing (Table 2).

### 4.2. Selected Sensor Characteristics

**Figure 3:**
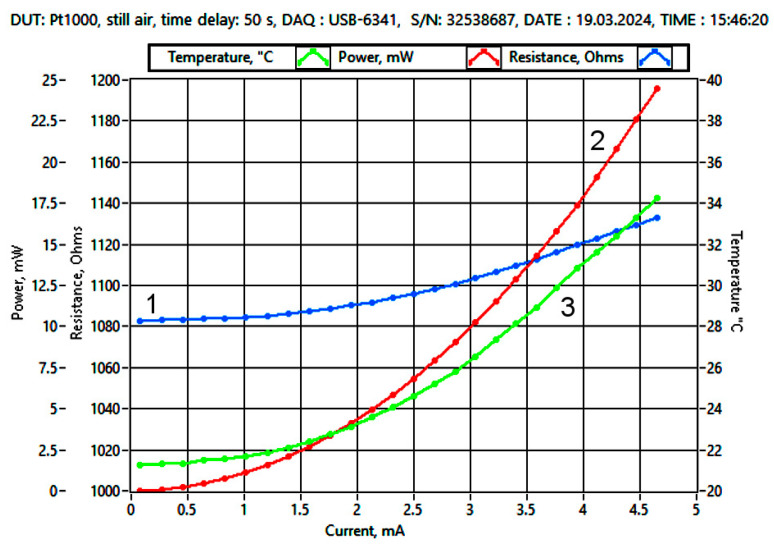
Characteristic of the Pt1000 sensor obtained from measurements: 1—resistance *R*_sens_ (blue), 2—heating power *P_th_* (red), and 3—sensor temperature *T*_sens_ (green), as a function of measurement current, delay time 50 s in still air. Least-squares approximation was used.

Figure 3 shows an example of the temperature dependence *T_sens_* (green) of a Pt1000 sensor (number 1) as a function of the measurement current. In addition, the graph shows a plot of sensor resistance *R_sens_* (blue) and sensor heating power *P_th_* (red). Each measurement was taken after a delay time of 50 s from the new set value of the current. The sensor reached a thermal steady state in still air.

**Figure 4:**
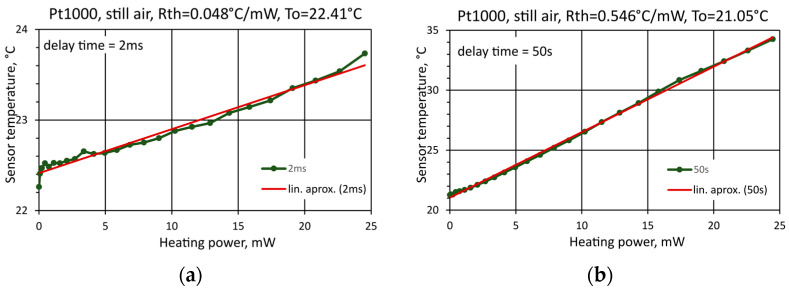
Linear approximation (red) by the least-squares method of the dependence of the temperature *T_sens_* (green) on the heating power *P_th_* for the Pt1000 sensor in still air; (**a**) measurement delay time of 2 ms; (**b**) measurement delay time of 50 s.

The resulting Pt1000 sensor temperature characteristics as a function of heating power are shown in Figure 4. It should be noted that the delay time, set as a parameter, between setting the new current value and measuring the sensor resistance was important for obtaining these results. In Figure 4a, this delay time is very short (2 ms), while Figure 4b shows measurement results for a much longer delay time (50 s). Note that the thermal resistance values obtained for the different delay times were significantly different. By approximating the measurement results with a straight line in the form: *y* = *a*_0_ *+ a*_1_*x*, we obtained the thermal resistance of the sensor *R_th_* = *a*_1_ and the ambient temperature *T*_amb_ = *a*_0_ (8). Least-squares approximation was used.

**Figure 5:**
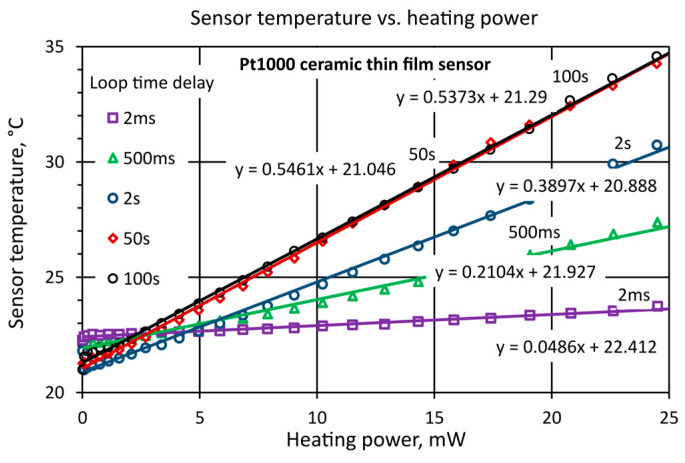
Linear approximation of temperature dependence of sensor *T*_sens_ with respect to heating power *P_th_,* delay time 2 ms, 500 ms, 2 s, 50 s, Pt1000 sensor in still air. The equation of the approximating straight line is shown.

The effect of measurement time duration on the value of the obtained thermal resistance *R_th_* was investigated in more detail for the Pt1000 sensor. Measurements were carried out for measurement loop delay times ranging from 2 ms to 50 s. Figure 5 shows examples of measurement results for the Pt1000 sensor in still air, for delay times of 2 ms, 500 ms, 2 s, 50 s. In Figure 5, it can also be seen that for longer delay times, the thermal resistance of the sensor *R_th_* increases significantly, from a value of 0.0486 °C/mW to 0.546 °C/mW, i.e., more than tenfold. Similar results were also obtained for other tested sensors. Depending on the delay time, significantly different values of the sensor’s thermal resistance *R_th_* were obtained. The change in *R_th_* values for all sensors was large, even reaching about tenfold when changing the delay time from 2 ms to 50 s. This can be explained by the occurrence of self-heating due to internal and external heat transfer effects in the sensor [2].

For short delay times, the thermal resistance is due to the internal effect in the sensor, while for longer times, it is due to the external effect in the sensor [2]. The effects observed in Figure 5 are related to the presence of the heat capacity of the sensors *C_th_* (Figure 1), which, together with the thermal resistance *R_th_* of the sensor, determines the rate of change of the sensor temperature.

Self-heating of the sensor due to the internal effect depends on the heat transfer inside the sensor, i.e., on its design, and does not depend on the heat exchange conditions between the sensor and the environment. On the other hand, the self-heating of the sensor caused by the external effect depends on two components. The first of these includes the conditions for heat transfer from the sensor sheath to the surrounding fluid. The second relates to the physical properties of the medium in which the sensor is placed and, most importantly, on the speed of movement of this medium.

### 4.3. Selected Characteristics of All Tested Sensors

**Figure 6:**
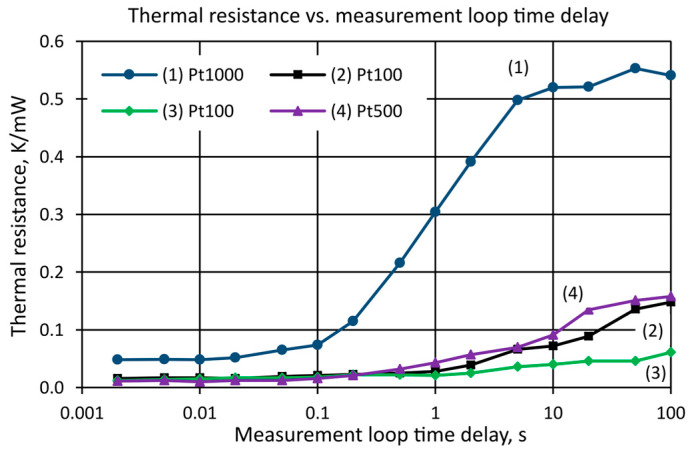
Thermal resistance of the sensors’ tested *R_th_*, depending on the delay time of the measurement loop.

The characteristics presented in Figure 2, Figure 3, Figure 4 and Figure 5 in Section 4.2 refer to one selected thin-film temperature sensor PT1000 (number 1). However, it should be noted that all the sensors were tested in the same way. Figure 6 shows the dependence of the thermal resistance of the sensors measured for different values of delay time in still air, stating the different values of *R_th_* for measurements made for a short time up to about 100 ms and for measurements made from 10 to 100 s.

For all the tested sensors, for times from 100 ms to about 10 s, the thermal resistance *R_th_* increased significantly. For sensor (1), the thermal resistance increased by about 10 times. For sensors (2), (3), (4), the increase was smaller at about 5 times. However, it should be noted that for all these sensors, the internal effect was practically the same. The Pt1000 (1) sensor was enclosed in a small case with a relatively small *S*_sens_ heat transfer surface, resulting in a relatively high thermal resistance. The other sensors (2), (3), (4) had large cases made of ceramic and metal, so their thermal resistance was much lower. Summarizing the results shown in Figure 6, it is reasonable to distinguish between the internal thermal resistance of the PRT sensors for measurements made in a short time (up to 100 ms) and the external thermal resistance for measurements made more slowly (longer than 10 to 100 s). For the sensors tested, the ratio of these two thermal resistances (internal and external) ranges from 5 to 10, depending on the sensor design.

Table 2 summarizes the numerical values of the thermal resistance *R_th_* obtained in the measurements, the temperature measurement errors Δ*T*_sens_ caused by the self-heating effect for the current *I*_sens_ = 1 mA, and the value of measurement current *I*_sens_ resulting in an error Δ*T*_sens_ = 1 °C. It should be remembered that the error Δ*T*_sens_ is proportional to the heating power of the *P_th_* sensor, and is therefore proportional to the square of the sensor current *I*_sens_. The small value of the internal thermal resistance *R_th_* allows measurements to be made in a short time at a higher current *I*_sens_ within the sensor and therefore, at a higher voltage drop across the sensor. This is advantageous because it allows more accurate measurements of the sensor’s electrical resistance *R*_sens_ and better noise immunity, and therefore, more accurate temperature measurements.

### 4.4. Measurement Uncertainty Evaluation

The final measurement result is complete only if it includes both the value of the measurement result and the uncertainty of the measurement result associated with that value. In the present study, the uncertainty values obtained by the Type A and Type B methods were determined. The procedure for calculating the uncertainty with the aforementioned methods for the USB-6341 module was as presented in previous publications [25,26]. Finally, based on knowledge of the uncertainty values determined by the Type A and B methods, the expanded uncertainty value *U* was determined for all the temperature sensors tested. During the experiments, for each sensor tested, the value of the reference resistor *R_N_* was selected equal to the initial resistance *R*_0_ of that sensor. During the sensor experiments, the temperature did not exceed 40 °C, meaning that the resistance of the sensors varied by no more than several % from the initial value. For this reason, when estimating the uncertainty of voltage measurements in both channels of the ADC, similar results were obtained for all sensors tested. Therefore, the estimation of voltage measurement uncertainty is presented for only one example, the Pt1000 thin-film temperature sensor. Type A uncertainty was computed as the standard deviation of the mean value. Finally, the combined uncertainty *u_c_* and the expanded uncertainty *U* = *k u_c_*, where the value of the coverage factor *k* = 3, were derived from the confidence level *p* = 99.73% recommended in the guidelines [27].

**Figure 7:**
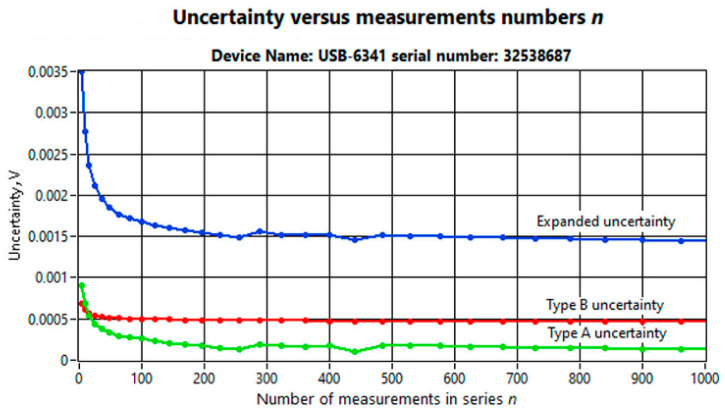
Uncertainties obtained from the measurements for NI USB-6341 module and Pt1000 sensor.

Since the Type A uncertainty depends on the number n of averaged measurements in the series, it is important to choose the number n accordingly. To this end, a series of experiments was carried out for values from *n* = 4 to *n* = 1000. Figure 7 shows the results of experiments carried out for a Pt1000 thin-film temperature sensor using the USB-6341 module. The graphs show the values of the evaluated Type A (green line), Type B (red line), and expanded uncertainty (blue line), as a function of the number of n measurements.

As can be seen in Figure 7, increasing the number of measurements beyond 100 was not very efficient, as it resulted in small values of changes in the expanded uncertainty. For this reason, in this study, the number of measurements in the series *n* = 100 was assumed. Making this assumption resulted in a value of the expanded uncertainty *U* for a voltage measurement of 1.7 mV. The uncertainty of the heating power measurement was calculated using the method of the law of propagation of uncertainty in indirect measurements, according to [27]. The corresponding partial derivatives of Relation (12) were calculated. The results of the uncertainty evaluation for the Pt1000 sensor are presented below. The limiting error ±Δ_mpe_ of the reference resistor *R_N_* = 1000 Ω was equal to 0.01%. For this resistor, the value of Type B uncertainty *u*_B_ was 58 mΩ, where *u*_B_ = ±Δ_mpe_/√3. Finally, the expanded uncertainty *U* of the heating power *P_th_* was 0.013 mW. Similarly, the expanded uncertainty of the temperature measurement was determined by calculating the corresponding partial derivatives from Relation (2), taking into account the relation for the resistance of the sensor *R*_sens_ (10). Finally, the expanded uncertainty *U* of the temperature measurement was 0.14 °C. The uncertainty in determining the sensors’ thermal resistance was determined by estimating the standard deviation of the directional coefficient of the straight line *y* = *ax* + *b* approximating the graph of the sensor temperature as a function of the heating power (Figure 2d), according to the relations presented previously [28,29]. The calculated value of the expanded uncertainty *U* of the *R_th_* thermal resistances sum for the Pt1000 sensor was not greater than 0.012 K/mW.

### 4.5. Discussion of the Results

From the graphs in Figure 6 and the measurement results shown in Table 1, it can be seen that the results of the thermal resistance of the *R_th_* sensors tested varied with the delay time in the measurement loop. For short delay times up to 100 ms, the thermal resistance of all the tested sensors had a lower value than the thermal resistance measured for long delay times greater than 10 s. The results of the thermal resistance measurements for short delay times were due to the self-heating effect. In this case, only the resistive element of the sensor (e.g., the platinum wire of an IPRT sensor) heated up and transferred the heat to the ceramic, glass, or epoxy resin sensor housing. However, because of the short delay time, there was no noticeable heat transfer between the sensor and its surroundings. In this situation, the internal thermal resistance of the sensor was measured.

It can be seen from Table 1 that most of the sensors tested had a small similar internal thermal resistance. This was due to the similar design of sensors 2, 3, and 4 (large housing, platinum wire resistive element). The exception was sensor 1 (a small sensor without any case, low mass of thin-film platinum resistive element), whose internal thermal resistance was about four times higher than the internal thermal resistance of sensors 2, 3, and 4. These results confirm the significant influence of housing size and the material from which it is made on the internal thermal resistance of the sensor.

The results of the thermal resistance measurements for long delay times were due to an external effect. In this case, there was significant heat transfer from the sensor to its surroundings. It is worth noting the significant influence of the type of sensor housing on the external thermal resistance measurement results. Sensors with larger housings (e.g., 2, 3, and 4) have lower external thermal resistance because the larger housing more easily transfers heat to the environment.

**Figure 8:**
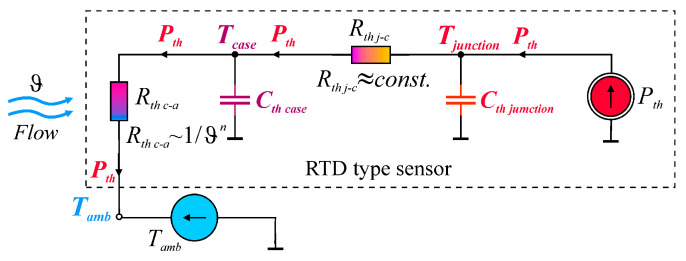
Equivalent diagram of the resistance sensor including internal and external thermal resistance.

It should be noted that the sensor block diagram shown in Figure 1 is too simplified to explain the effects observed in the measurements. In this schematic, it is not possible to distinguish between internal and external effects. Therefore, in Figure 8, an extended version of the schematic in Figure 1 is presented. The thermal resistance *R_th_* has been separated into two components: the internal and external thermal resistance. Similarly, the heat capacity *C_th_* has been divided into two components, which are the heat capacity *C_th junction_* of the resistive sensor element itself and the heat capacity *C_th case_* of the sensor case. It is worth noting the symbols used in Figure 8. The thermal resistance discussed is also an important parameter to consider for semiconductor components such as rectifier diodes, LEDs, transistors, thyristors, etc. For these semiconductor devices, the concept of thermal resistance between the junction and the case *R_th j-c_* and the thermal resistance between the case and the ambient *R_th c-a_* is common. It should be noted that the resistance *R_th j-c_* is due to the internal effect, and the resistance *R_th c-a_* is due to the external effect. For this reason, the equivalent diagram shown in Figure 8 does not introduce new designations for thermoresistive sensors, but the familiar and commonly used in industrial electronics designations for the internal thermal resistance *R_th j-c_* and for the external thermal resistance *R_th c-a_* are used.

The same approach can be applied to more complex structures such as integrated circuits, discrete power components, multiprocessor modules, LEDs, transformers, and even large telecommunications systems. In these types of systems, the internal resistance of the *R_th j-c_* characterizes the thermal path from the active part inside the device, through the materials used to support and bond the die to the case or outer surface of the chip package. The external resistance *R_EX_* is the resistance associated with the transfer of heat from the case directly to the coolant or indirectly through a heat sink or finned structure. The thermal resistance *R_th c-a_*, on the other hand, refers to the thermal resistance of the case to the ambient surroundings. For fixed power dissipation, low values of *R_th j-c_* and *R_th c-a_* result in low die temperatures and are preferred under nearly all circumstances [30]. For temperature sensors, on the other hand, the small values of these thermal resistances ensure small errors in temperature measurement due to the self-heating effect.

## 5. Conclusions

When SPRT standard sensors are used, a calibration process is performed with the same measurement current at which the sensor is to be used for measurements. For high-accuracy measurements, it is necessary to carry out error correction with regard to the sensor’s self-heating. For this group of sensors, manufacturers usually provide values for self-heating ratios in the specific medium in which they will be used during operation. This paper focuses on a group of industrial IPRT sensors that are often used in practice, but the effect of self-heating on these sensors has been described sporadically in the literature. It is recommended that the value of the measuring current is selected in such a way that the effect of self-heating of the sensor does not exceed 25% of the tolerance value resulting from the declared tolerance of the sensor. However, at the same time, a higher measuring current ensures a higher accuracy of the sensor resistance measurement. Therefore, in order to correctly select the measurement current of the IPRT sensor, the in situ thermal resistance of the sensor must be determined. The value of this parameter should be assessed under the specific operating conditions of the sensor, i.e., for the medium in which the sensor operates and its flow conditions. According to the authors, for industrial temperature sensors, this issue has so far not been sufficiently addressed in the literature, while for standard sensors, the literature on the study of the self-heating effect is extensive. The standards for IPRT sensors define the concept of a self-heating coefficient, which is equivalent to the concept of thermal resistance. Methods for determining the thermal resistance of sensors under well-defined conditions are also described, as well as the requirements that these sensors should meet in order to reduce the self-heating effect to an acceptable level. This makes it possible to compare the properties of sensors from different manufacturers with each other. For the user, however, this information is of little use in most cases. This is because, in a specific industrial application, the sensor usually operates under conditions completely different to those described in the standards. The way in which the sensor is mounted and its operating conditions fundamentally influence the self-heating effect and the thermal resistance of the sensor. This is why the technical data of the sensor (in particular, the thermal resistance) provided by the manufacturer and determined in accordance with the standard are of no use to the user. To use the sensor correctly and in particular, to select the value of the measuring current accordingly, the thermal resistance of the sensor must be tested and determined in situ.

This paper discusses the problem of self-heating resistance sensors and its impact on the reliability of temperature measurement. Several industrial resistance sensors, commonly used in practice, were used in the study. The authors’ LabVIEW application, developed and presented in this paper, enables automatic in situ measurements of the sensor’s thermal resistance. Test results are reported in the form of graphic files and numerical data. The reports prepared contain information about the measurement module used, the type and parameters of the sensors, as well as the date and time when the tests were performed. The presented thermal resistance values cannot be applied to other sensors operating under other conditions. The main result of the work is the development of a method and a system for the in situ experimental determination of the thermal resistance of metallic thermoresistive sensors (platinum, nickel, Balco, copper). The presented method also enables the study of the self-heating effect for other objects, e.g., NTC, PTC thermistors, tungsten light bulb filaments, thermoanemometric sensors, copper wires in electrical installations, etc. The solution presented in this paper enables optimal selection of power supply conditions for thermoresistive sensors, depending on their design, environmental conditions, and the way the sensor is mounted. An important parameter in the in situ determination of the thermal resistance of a sensor is the delay time between the assignment of a new measurement current value and the measurement of the electrical resistance of the sensor. Changing the value of this time significantly affects the results obtained from the thermal resistance measurement. If the delay time is short (up to 100 ms), the measurement results represent the internal effect in the sensor. This is due to the heat capacity of the sensors, which causes the sensor temperature to rise too slowly. If a much longer delay time is selected (more than 10 s), a thermal resistance is obtained that represents the external effect on the sensor. Measurements show that the external thermal resistance is two to ten times greater than the internal resistance, depending on the sensor type. At the same time, the external thermal resistance strongly depends on the parameters of the medium in which the sensor is placed, in particular, on the velocity of the fluid surrounding the sensor.

Therefore, it is worth noting that the correct application of the sensor is influenced by the external effect, which depends on how the sensor is mounted and the parameters of the surrounding medium. Therefore, the authors consider it appropriate to carry out the study of the self-heating effect of industrial sensors as presented in this paper using the in situ method. The small value of the internal thermal resistance allows measurements to be made with a short pulse of high current. This is possible because of the large heat capacity of the sensor when the amount of heat delivered in a short current pulse is sufficiently small. This does not result in a significant increase in the temperature of the sensor, and the measurement error remains sufficiently small. At the same time, this solution for portable measuring devices can significantly reduce power consumption. Determining the thermal resistance of the sensor also allows one to measure the flow rate of the fluid surrounding the sensor. For this purpose, the dependence of the thermal resistance of the sensor on the fluid flow rate needs to be determined experimentally. This issue will be the subject of further work by the present authors.

## Figures and Tables

**Table 1:** The parameters of NI USB-6341 module used for the measurements [25].

Range V_Diff_	Gain Error ppm of Reading	Offset Error ppm of Range	Offset Tempco ppm of Range/°C	Random Error μVrms	Absolute Accuracy at Full Scale mV
±10	65	13	23	270	2.190

**Table 2:** Summary of the obtained test results for the parameters of the tested sensors in still air.

Symbol	Case	Internal *R_th_* K/mW	External *R_th_* K/mW	Δ*T*_sens_@1 mA (External) °C	*I*_sens_@ Δ*T*_sens_ = 1 °C (External) mA
Pt1000	thin film	0.048	0.553	0.553	1.35
Pt100	ceramic	0.016	0.148	0.015	8.22
Pt100	steel industrial case	0.012	0.061	0.006	12.8
Pt500	steel laboratory sensor	0.011	0.158	0.079	3.56

## Data Availability

All calculated, measured data and the program of LabVIEW will be provided upon request to the correspondent authors by email, with appropriate justification.

## Abbreviations

The following abbreviations are used in this manuscript:

AWS	Automatic weather station
IPRT	Industrial platinum resistance thermometer
PRT	Platinum resistance thermometers
RTD	Resistance temperature sensors
SPRT	Standard platinum resistance thermometers
